# Fisheries regulatory regimes and resilience to climate change

**DOI:** 10.1007/s13280-016-0850-1

**Published:** 2016-11-16

**Authors:** Elena Ojea, Isaac Pearlman, Steven D. Gaines, Sarah E. Lester

**Affiliations:** 1 Future Oceans Lab, University of Vigo, Edificio Torre CACTI, Campus Universitario, 36310 Vigo, Spain; 2 Basque Center for Climate Change (BC3), Bilbao, Spain; 3 Bren School of Environmental Science & Management, University of California, 2400 Bren Hall, Santa Barbara, CA 93106-5131 USA; 4 Department of Geography, Florida State University, Bellamy Building, Tallahassee, FL 32306-2190 USA

**Keywords:** Climate change adaptation, Fisheries systems, Resilience, Socio-ecological systems

## Abstract

Climate change is already producing ecological, social, and economic impacts on fisheries, and these effects are expected to increase in frequency and magnitude in the future. Fisheries governance and regulations can alter socio-ecological resilience to climate change impacts via harvest control rules and incentives driving fisher behavior, yet there are no syntheses or conceptual frameworks for examining how institutions and their regulatory approaches can alter fisheries resilience to climate change. We identify nine key climate resilience criteria for fisheries socio-ecological systems (SES), defining resilience as the ability of the coupled system of interacting social and ecological components (i.e., the SES) to absorb change while avoiding transformation into a different undesirable state. We then evaluate the capacity of four fisheries regulatory systems that vary in their degree of property rights, including open access, limited entry, and two types of rights-based management, to increase or inhibit resilience. Our exploratory assessment of evidence in the literature suggests that these regulatory regimes vary widely in their ability to promote resilient fisheries, with rights-based approaches appearing to offer more resilience benefits in many cases, but detailed characteristics of the regulatory instruments are fundamental.

**Electronic supplementary material:**

The online version of this article (doi:10.1007/s13280-016-0850-1) contains supplementary material, which is available to authorized users.

## Introduction

Marine systems have been and will continue to be impacted by climate change across all habitats, latitudes, and trophic levels (Hoegh-Guldberg and Bruno 2010; Richardson et al. 2012; Poloczanska et al. 2013; Rhein et al. 2013). These impacts trickle up to the fisheries that humans depend upon via alterations in primary productivity (Poloczanska et al. 2013), food webs (Edwards and Richardson 2004), and community structure (Barange and Perry 2009). These ecological shifts will produce both positive and negative impacts (Cheung et al. 2012), but are expected to affect fishers and fishing communities via altered fishing revenues, increased costs per unit effort, higher insurance costs, and/or changes in international fishing agreements, among others (Adger et al. 2009; Badjeck et al. 2010; Sumaila et al. 2011). Thus, there is a need for fisheries management that promotes resilient social and ecological systems in order to ensure long-term sustainability (Adger et al. 2009; Grafton 2009).

While ecological resilience is traditionally defined as the *resistance* of ecosystems or species to disturbance, and the speed of *recovery* following disturbance (Holling 1973); in social systems resilience is related to the disturbance, reorganization, and renewal of communities and institutions (Grafton 2009). Here we define socio-ecological resilience as the ability of a SES to absorb disturbances while retaining the same basic structure and ways of functioning, the capacity for self-organization, and the capacity to adapt to stress and change (IPCC 2007, Davidson et al. 2013). Social–ecological resilience refers to people and nature as inter-dependent systems (Folke et al. 2010), where changes in ecological resilience affect social resilience and vice versa.

We define a SES as a coupled and integrated system that encompasses social and ecological components that interact closely (López-Angarita et al. 2014). Fisheries SES are regulated in diverse ways including community management, co-management, and strong top-down control rules (e.g., limited licensing of vessels). Fisheries regulatory regimes also vary in the extent of rights allocated to fishers (e.g., access rights, property rights) and regulations stemming from conservation measures that might be in place (e.g., marine protected areas). These institutional and regulatory characteristics can have important implications for how a fishery SES responds to climate change. For example, protecting particular age classes by using more selective gear (Cinner et al. 2009b; Grafton 2009) or protecting key habitat by implementing no-take marine reserves (Allison et al. 2003; Gaines et al. 2010) can buffer stocks against climatic shocks, helping fishers and communities to adapt. Similarly, promoting long-term resource stewardship, for instance via property rights, may provide incentives to fishers to apply more precautionary or adaptive management approaches in the face of looming climate change.

Many fisheries regulatory strategies may be ill prepared to protect or enhance resilience to climate change (McClanahan 2008). There is a growing body of research that addresses the question of resilience to climate change from different angles: by looking at climate change adaptation and resilience in specific fisheries SES (Cinner et al. 2009a; Lopes et al. 2011; Pinsky and Mantua 2014; Maldonado and Moreno-Sánchez 2014) by assessing the adaptive capacity of fisheries SES to confront climate change (Leith et al. 2014; López-Angarita et al. 2014; Rivera et al. 2014); and by identifying attributes that safeguard economic (van Putten et al. 2013) and ecological resilience for a given fishery (McClanahan et al. 2012). However, no studies have suggested broadly applicable criteria for assessing socio-ecological resilience of fisheries. Moreover, the implications of different regulatory systems for fisheries’ resilience to climate change is yet unknown (Smit et al. 2001; Folke 2006; Pinsky and Mantua 2014).

This paper addresses this gap by conceptualizing resilience in fisheries management systems by identifying nine resilience criteria. We then explore four fisheries regulatory systems (individual transferable quotas—ITQs; territorial use rights for fisheries—TURFs; limited entry; and open access) in terms of their capacity for social and ecological resilience to climate change based on these criteria, examining evidence in the literature. Our aim is to provide a framework for understanding the effects of fisheries regulatory regimes under a resilience perspective. Our preliminary assessment of the resilience potential of these regulatory approaches suggests important research directions to promote fisheries able to adapt to impending climate change.

## Methodological approach

We derive three ecological criteria and six social criteria for fisheries resilience from the literature, and conceptualize the interactions among these criteria in terms of achieving overall resilience for fisheries SES. We follow a step-wise process where we (1) identify the main criteria from the scientific literature that have been proposed and/or observed to contribute to ecological and social resilience in fisheries systems; (2) discuss the potential of the selected fisheries regulatory regimes to achieve the resilience criteria; and (3) illustrate with available examples evidence of the regulatory regimes supporting or compromising each resilience criterion.

We focus the analysis on four classes of fisheries regulatory systems (TURFS, ITQs, limited entry, and open access) in terms of their potential to promote or inhibit resilience to climate change. We select these management systems based on different levels of access privileges for individuals and/or groups (Hilborn et al. 2005). Here open access represents the least exclusive regime in which anyone who wishes can participate. Under limited entry, licenses allow for the right to participate in fishing the resource contingent on compliance with regulations such as gear and/or effort limitations. In ITQs, quota ownership is required to fish a proportion of the total catch or effort, and finally, in TURFS, rights are granted to fish specific fishing grounds, an approach that is somewhat more common in artisanal and small-scale fisheries (Hilborn et al. 2005). We focus on these regulatory regimes because there has been a lot of interest in the literature on the benefits of more exclusive access for fisheries (see Costello et al. 2008 for example), but our framework could also be applied to other types of fisheries governance and management. See the 10.1007/s13280-016-0850-1 (Methods section) for a more detailed description of the steps and methods followed.

## Climate resilience criteria

From a detailed review of the literature, we identify three ecological climate resilience (ECR) and six socio-economic climate resilience (SCR) criteria for assessing the resilience potential of fisheries management systems. We summarize these criteria in Table 1, where we include the rationale supporting each criterion and references that support the link between resilience and the specific criterion. A more detailed description of the different criteria and supporting literature is available in 10.1007/s13280-016-0850-1.

**Table 1:** Fisheries socio-ecological climate resilience criteria

Climate resilience criteria	Rationale	Literature sources
*ECR*-*1: sustainable & age*-*diverse target populations*	Increased population abundance, age structure, and genetic diversity buffer against stock collapse from environmental shocks	Brander (2009), Perry et al. (2010), and Hsieh et al. (2006)
*ECR*-*2: conserving biodiversity & habitats*	Conserving biodiversity, community structure, and habitats support fish population resistance and recovery to external stressors	Levin and Lubchenco (2008) and Worm et al. (2006)
*ECR*-*3: managing existing stressors*	Non-climatic stressors (e.g., pollution, habitat destruction) render fisheries systems less resilient to climate impacts	Crain et al. (2008), Feely et al. (2010), and Cai et al. (2011)
*SCR*-*1: adaptive management*	Institutional capacity to experiment and learn are necessary to cope with uncertain and unforeseen climate impacts on fisheries	de Moor et al. (2008), Plaganyi et al. (2011), Davidson et al. (2013), and Rivera et al. (2014)
*SCR*-*2: diversified livelihoods*	Alternative sources of income for fishers increase social resilience in the face of economic instability from climate impacts on fisheries	Allison and Ellis 2001, Sumaila et al. (2011), Grafton (2009), and Badjeck et al. (2010)
*SCR*-*3: promoting long*-*term stewardship*	Promoting long-term stewardship provides incentives to manage the resources sustainably in the face of future climate impacts	Essington et al. (2012), Cancino et al. (2007), Jardine and Sanchirico (2012), and Nowlis and van Benthem (2012)
*SCR*-*4: multi*-*level governance*	Governance over a fishery at different scales creates a flexible structure for adapting to change at multiple scales	Grafton (2009), Hughes et al. (2005), and Fidelman et al. (2013)
*SCR*-*5: Fisher mobility*	Technology and capacity to change fishing locations increase social resilience under shifting stocks	Sumaila et al. (2011), Grafton (2009), and Pinsky and Mantua, (2014)
*SCR*-*6: community*-*based management*	Community-based management can improve economic conditions for fishers and mitigate environmental impacts	Tompkins and Eakin (2012), Adger (2003), Ovando et al. (2013), and Defeo et al. (2014)

*ECR* ecological resilience criteria, *SCR* socio-economic resilience criteria

**Fig. 1:**
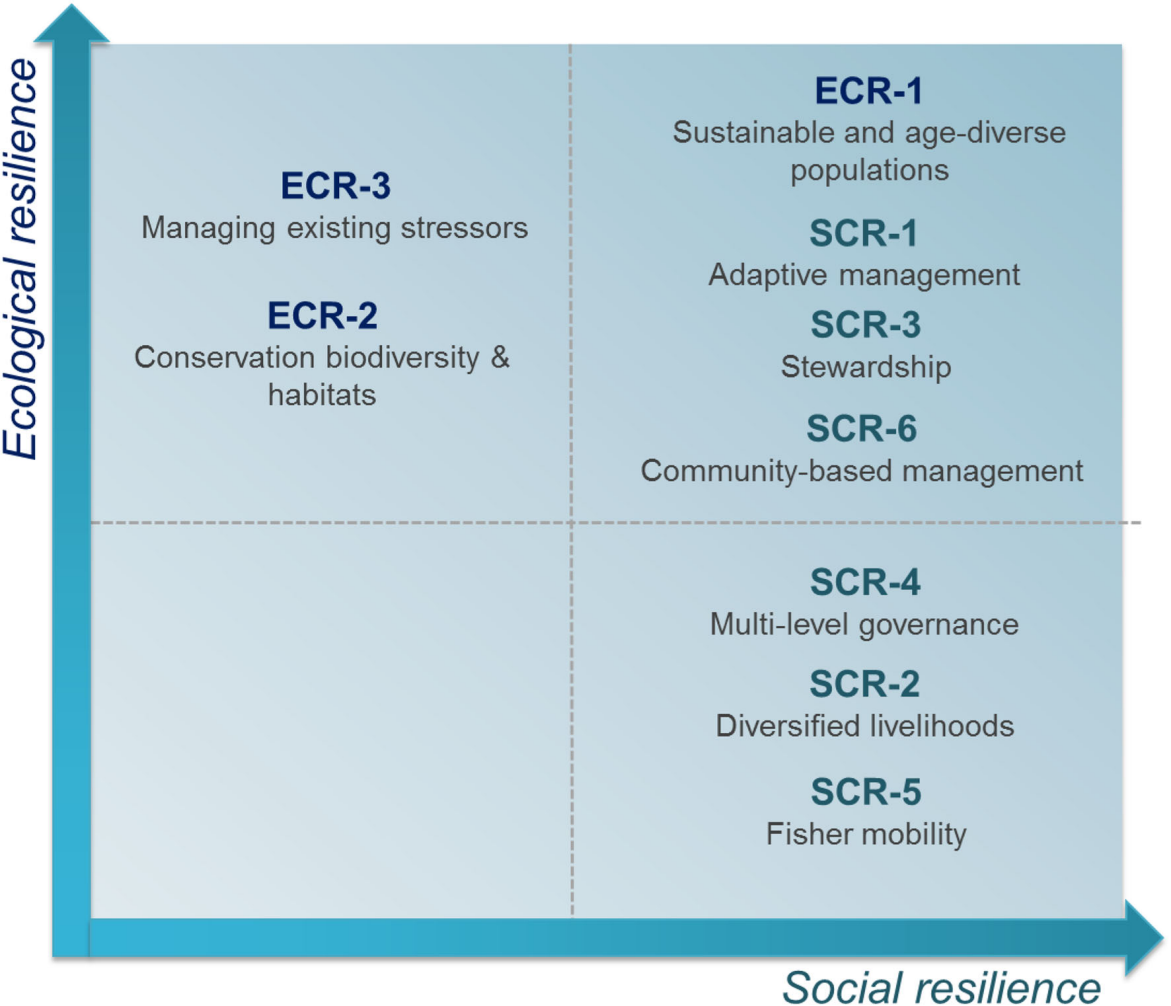
Tradeoffs and synergies in socio-ecological resilience criteria for fisheries. *ECR* ecological criteria, *SCR* social criteria

Although we classify resilience criteria as either social or ecological, feedbacks and interactions exist whereby ecological criteria can enhance or erode social resilience, and vice versa, creating complex tradeoffs and synergies within fisheries’ resilience options. Based on arguments made in the literature, the nature of these social–ecological resilience interactions for the nine criteria is represented in Fig. 1.

Ecological criteria like Sustainable and age-diverse populations (ECR-1) and Conservation of biodiversity and habitats (ECR-2) have clear ecological benefits for resilience (Levin and Lubchenco 2008; Perry et al. 2010; Bernhardt and Leslie 2013), which can also enhance social resilience as fishers may have a more secure source of income when fish populations are sustainably managed (ECR-1). These social benefits can also arise in the longer run as habitats and ecosystems are in a good state (ECR-2). Managing existing stressors (ECR-3) is expected to increase ecological resilience (Barange and Perry 2009), but can have varying effects for social resilience. For example, decreasing stressors like pollution may increase revenues due to an increase in quality/quantity of fish, but can also compromise livelihood diversification through more restrictive regulations placed on other sectors (i.e., agriculture, Badjeck et al. 2010).

Social criteria, specifically adaptive management (SCR-1) is likely to increase ecological resilience by incorporating learning and new science into management (Plaganyi et al. 2011; Rivera et al. 2014), which can increase the likelihood that fishing pressure is sustainable and prepare fishers for abrupt and unexpected changes (Lehodey et al. 2006). Diversified livelihoods (SCR-2) may promote social resilience by decreasing reliance on fisheries (Cinner et al. 2009a; Defeo et al. 2014; Pinsky and Mantua 2014), but the impacts on ecological resilience remain unclear as fishers may engage in other activities that are damaging for the ecosystem, and/or lack stewardship incentives if they can easily exit the sector. Stewardship incentives (SCR-3) can increase ecological resilience as fishers may advocate for sustainable management. Stewardship is promoted under long-lasting access to the resource, which also contributes to social resilience (Essington et al. 2012). Multi-level governance (SCR-4) increases social resilience by providing flexibility in resource management, for example by matching management scales to biological scales of the resources. Multi-level governance can also facilitate the implementation of adaptive management that increases ecological resilience (Hughes et al. 2005). The Maine lobster fishery provides a good example of multi-level governance that led to careful monitoring, adaptive management, and sustainable harvest of the resource (Schultz et al. 2015).

Fisher mobility (SCR-5) can increase social resilience, especially for industrial fisheries (Pinsky and Mantua 2014) that have the capacity to change fishing grounds easily (Allison and Ellis 2001). For small-scale fisheries, mobility can be also be beneficial for accessing new areas that become more productive due to shifting stocks. In both cases, fisher mobility may decrease ecological resilience as fishers can deplete one area and move to the next, but may increase social resilience as fishers access new fishing grounds. This potential positive effect of mobility for social resilience could be limited, however, by negative interactions among ‘original’ and ‘new’ fishers in a given area. Finally, Community-based management (SCR-6) is expected to increase social resilience by enhancing the sense of stewardship and sensitivity of fishers to socio-economic constraints, and by allowing for the incorporation of local knowledge of the resource (Gutierrez et al. 2011). Community-based management is also a potentially useful approach to generate adaptive capacity (Rivera et al. 2015). Adaptive capacity refers to the ability to anticipate and respond to disturbances, and to minimize, cope with, and recover from their consequences (Maldonado and Moreno-Sánchez 2014). Community-based management has also been shown to improve compliance with rules (e.g., catch limits, size limits), monitoring, and surveillance (Gutierrez et al. 2011) that will directly benefit ecological resilience.

## Climate resilience of fisheries regulatory regimes

Fisheries regulatory systems vary in their approaches to harvest control rules, fisher behavioral incentives, and adaptability to a changing environment. We examine specific regulatory approaches ranging from open access to limited entry to rights-based systems (ITQs and TURFs), and discuss their likelihood of meeting the above resilience criteria. To illustrate the potential effects of regulatory regimes on fisheries resilience, we collect a number of examples from the literature that we describe here and summarize in Table 2.

**Table 2:** Examples in the literature of regulatory regimes leading to (+) or compromising (−) the resilience criteria (OA—Open Access, LE—Limited Entry, ITQ—individual transferable quotas, TURF—Territorial Use Rights for Fisheries)

	OA	LE	ITQ	TURF
*ECR*-*1: sustainable & age*-*diverse target populations*	− EU CFP (Hentrich and Salomon 2006)	− New England Groundfish (Hilborn et al. 2005) + Bristol Bay pacific salmon (Hilborn et al. 2003)	+ New Zealand lobster fishery (Hilborn et al. 2005)	+ Chilean TURFs (Hilborn et al. 2005)
*ECR*-*2: conserving biodiversity & habitats*	− US west coast trawling (Hilborn et al. 2005)	− New England groundfish (Hilborn et al. 2005) + Eastern Australia longline fishery (Hobday et al. 2010)	+ US Ocean quahog and surf clams fisheries (Arnason 2002) − US West coast groundfish (Hilborn et al. 2005)	+ Chilean TURFS (Gelcich et al. 2012)
*ECR*-*3: managing existing stressors*	− Canadian northern cod fishery (McCay et al. 2011)	− US west coast trawl (Hilborn et al. 2005)	+ New Zealand ITQs (Hentrich and Salomon 2006)	+ Asturias Goose Barnacle (Rivera et al. 2014)
*SCR*-*1: adaptive management*	− Canadian northern cod fishery (McCay et al. 2011)	− Galapagos sea cucumber fishery (Defeo et al. 2014)	+ New Zealand Chatham rise orange roughy (Hilborn et al. 2005)	+ Asturias Goose Barnacle (Rivera et al. 2014) + Chilean TURFs (Hilborn et al. 2005)
*SCR*-*2: diversified livelihoods*	− Kenyan coral reef fishery (Cinner et al. 2009a)	+ Galician artisanal fisheries (Allison and Ellis 2001)	− New Zealaland ITQ fisheries (Stewart et al. 2006)	+ Chilean TURFs (Moreno and Revenga 2012) + Asturias Goose barnacle TURF (Rivera et al. 2014)
*SCR*-*3: promoting long*-*term stewardship*	− Alaska fleet overcapacity in the 90s (Sissenwine and Rosenberg 1993)	− New England Groundfish (Hilborn et al. 2005) − Galician spider crab artisanal fishery (Freire et al. 2002)-	+ North Pacific Halibut/Sablefish fisheries (Arnason 2002)	+ Punta Allen spiny lobster TURF (Defeo et al. 2014)
*SCR*-*4: multi*-*level governance*	− US west coast trawl (Hilborn et al. 2005)	+ Great Barrier Reef Industrial fisheries (Fidelman et al. 2013)	+ Netherlands ITQ system (Hentrich and Salomon 2006)	+ Asturias goose barnacle TURF (Rivera et al. 2014)
*SCR*-*5: Fisher mobility*	+ Kenyan coral reef fishery (Cinner et al. 2009a)	+ Irish Sea and English Channel new anchovy stocks (Cheung et al. 2012)	− Argentinian hake (Hilborn et al. 2005)	− Chilean TURFS (Aburto et al. 2013) − Surf clam Chile (Aburto et al. 2014)
*SCR*-*6: community*-*based management*	− Malawi artisanal fisheries (Blaikie 2006)	+ US hake and pollock cooperatives (Hilborn et al. 2005) − Galapagos sea cucumber fishery (Defeo et al. 2014) − Galician spider crab artisanal fishery (Freire et al. 2002)	+ Tasmanian lobster fishery (van Putten et al. 2013)	+ Galician TURFs (Macho et al. 2013) + Punta Allen spiny lobster TURF (Defeo et al. 2014) + Asturias Goose barnacle TURFs (Rivera et al. 2014)

### Open access

Open access fisheries do not have well-defined access rights; access rights either are completely absent or are not enforced (Sumaila 2012). Under open access, the fish stock is rival but non-excludable, meaning that the right to fish is accorded to anyone, sometimes with a license or a nominal fee accessible to anyone. Individuals have incentives to maximize their profits, even at the expense of capturing as many fish as possible in a short time (Hardin 1968; Costanza et al. 1998), which can lead to unsustainable harvest levels and destructive fishing practices that will eventually erode the economic value of the fishery as well (Worm et al. 2009). Additionally, unlimited access can create a competitive ‘race to fish’ to catch fish before others do, which further exacerbates overfishing and incentivizes over-capitalization, redundant effort, and inefficient timing of harvest, again lowering the profitability of the fishery (Pauly et al. 2002; Costello et al. 2008, 2010). Many fisheries around the world are managed as open access; however, open access systems have a poor record of performance in terms of sustainability (Gordon 1954; Costello et al. 2010) and are a primary contributor to the stock collapse of many of the world’s fisheries (Pauly et al. 2002). In addition, for overexploited fisheries, reducing fishing pressure is the principal means of reducing the impacts of climate change (Brander 2007), and this may be difficult to achieve under open access regimes.

In open access fisheries, harvest decisions are often made with limited consideration of ecological information or economic costs and benefits to society (Costanza et al. 1998), and there is often low compliance with regulations due to the lack of well-defined access rights and enforcement (Sumaila 2012). An open access system lacks allocation of property rights, which have been demonstrated to create incentives for conserving biodiversity and habitats. Thus, in the absence of rights, open access does not promote the conservation of habitats and ecosystems (ECR-2), nor sustainable management (ERC-1), and intensive and sometimes destructive fishing prevails over sustainable use of the resource (Grafton et al. 2006). Well-known fisheries collapses have occurred in open access systems, like the Canadian Atlantic cod (McCay et al. 2011), in part because adaptive management (SCR-1) is discouraged by the regulatory regime.

Large open access systems may also experience complex stakeholder and institutional networks (Fidelman et al. 2013). For example in the Peruvian anchoveta, formal institutions are slow in responding to climate changes (Badjeck et al. 2009), constraining effective multi-level governance (SCR-4), and making community-based management (SCR-6) more difficult (Badjeck et al. 2009). In fact, management involvement of stakeholders who do not have a long-term interest in the fishery may even have negative effects on sustainability (Botsford et al. 1997), as they may lack stewardship incentives (SCR-3) (Costello et al. 2008). In theory for open access, fishers respond to profitability, entering a fishery depending on the expected economic returns at a given time. In this context, fishers enter and exit the fishery more freely, which may help them buffer against stock fluctuations or declining stocks by having developed alternative sources of income (livelihood diversification (SCR-2)) (Allison and Ellis 2001). However, this flexibility may be limited by the high capital investments in many open access systems that make it more difficult to exit the fishery, and for the poorest fishers, who may remain in the fishery despite severely declining stocks (e.g., Cinner et al. 2009a). Fisher mobility can be high in open access systems, and fishers may be accustomed to shifting from one area to another in the search for productive stocks (SCR-5) (Sumaila et al. 2011). On the ecological side, however, fisher mobility is related to a decrease in ecological resilience due to the serial depletion of stocks and to a lower capacity to monitor and determine population dynamics, making adaptive management more difficult (SCR-1) (DeYoung et al. 2008; Leith et al. 2014).

### Limited entry

Limited entry, also known as restricted access, occurs when a management institution establishes conditions (e.g., licenses, gear limitations, area/seasonal restrictions) that determine and limit who is able to fish (Townsend 1990). Most commonly in limited access, there are a fixed number of licenses issued that permit access to harvest the resource, as is the case for many commercial fisheries around the world (Hilborn et al. 2005).

Despite the different possible restrictions on access, fisheries can underperform or fail in economic and biological terms due to excessive capital investment (Hilborn et al. 2005), redundant effort, inefficient timing of harvest, or other inefficiencies that are also common to the open access regime (Costello et al. 2010). Indeed, failure to control effort can still lead a limited entry fishery to near open access overcapacity and effort conditions, with the subsequent negative implications for recovery under climate change (Brander 2007). This overcapacity provides no incentives to fishers to engage in sustainable management (Hilborn et al. 2005; Defeo et al. 2014).

Some limited entry fisheries have resulted in sustainable harvest (ECR-1) (Townsend 1990, Hilborn et al. 2003), generally when institutional systems provide incentives to individual operators to motivate sustainable harvest (Hilborn et al. 2005). However, solely restricting access in order to manage fisheries does not guarantee sustainable harvest or conservation of biodiversity or habitat (ECR-2) (Hilborn et al. 2005). Limited entry per se does not incentivize fishers to respond to fishery stressors (ERC-3) and avoid discards, for example, as evidenced in the US west coast trawling fishery that discarded 40 percent of catches (Hilborn et al. 2005). Successful examples exist, however, under mandatory controls to avoid by-catch, such as the East Australian longline fishery that uses limited entry spatial zones to conserve Bluefin tuna (Hobday et al. 2010). In regard to social resilience, limited entry as a management system usually fails to address stewardship (SCR-3) due to the low level of access rights, but may be flexible for engaging in alternative livelihoods (SCR-2) for the same reason (Allison and Ellis 2001). The top-down nature of restricting access frequently hinders community-based management (SCR-6), and in some cases also produces a mismatch between management and biological scales, as in the example of the Atlantic tuna fishery (Berkes 2006). A detailed analysis of effective multi-level governance (SCR-4) has been conducted in the Australian Great Barrier Reef fisheries (Fidelman et al. 2013). In this region, the potential of a multi-level approach to help the system adapt to climate change appears to be threatened by a complex and fragmented governance system (Fidelman et al. 2013).

Unlike open access, limited entry systems could be implemented more effectively from a community-based basis (SCR-6), as shown by hake and pollock cooperatives in the United States (Hilborn et al. 2005). However, community-based management within a limited entry setting may not be sufficient for achieving sustainable use of the resource, as in the case of the Galapagos sea cucumber fishery (Defeo et al. 2014), where the fishery was incapable of implementing adaptive management (SCR-1). In contrast, limited access regulations can be relatively easily adjusted—including changing the number or cost of licenses, or altering area and gear restrictions based on seasonal projections (Wilen 1988; Townsend 1990). This flexibility can potentially be beneficial for adaptive management (SCR-1), and for allowing fishing in new areas (SCR-5) when stocks shift their distributions (Cheung et al. 2012).

### Individual transferable quotas (ITQs)

Catch share systems such as individual transferable quotas (ITQs) allocate to owners the right to harvest a specified proportion of the total allowable catch (TAC), where rights are divisible, leasable, and transferable among users (Costello et al. 2010). Fish stocks are usually managed at a regional level where the responsible institution sets the TAC with which users must comply. ITQs are typically non-spatial, in the sense that stocks are often mobile and participants can harvest these stocks anywhere as long as they operate within agreed upon jurisdictional limits. This flexibility can help in adapting to climate change shifting stocks, but past examples have shown how unanticipated environmental regime shifts, such as those expected from climate change, pose serious challenges for maintaining international cooperation for transboundary stocks under ITQs, as for example the Pacific salmon conflict between the US and Canada (Miller and Munro 2004).

ITQs vary in design (e.g., tenure length, rules regarding transferability), which may impact resilience. For example, ITQs have been observed to decrease variability in landings and exploitation rates for multi-species fisheries, but this effect is only present when the ITQ right is durable and secure (Essington et al. 2012), namely when rights have an appropriately long tenure. In fact, secure rights have also been shown to provide higher asset values from the fishery (Grainger and Costello 2011). It is widely recognized that ITQ fisheries are less likely to experience a collapse in landings or to have excessive overfishing, and they typically lead to better compliance with catch limits compared to the absence of rights (Townsend 1990; Worm et al. 2006; Costello et al. 2008, 2010; Essington et al. 2012; Melnychuk et al. 2012; van Putten et al. 2013). Well-designed ITQs can lead fishers to support lower TACs (Costello et al. 2010), and to avoid short-term resource degradation (Essington et al. 2012). Long-term rights can also in some cases promote the conservation of non-target species or habitats (ECR-2). One example is fishers voluntarily retiring bottom longlines known to damage corals (Arnason 2002). However, when ITQs are allocated only to target species, there may be little incentive to avoid by-catch of non-target species, and therefore multi-species ITQs have been created to diminish by-catch discards (Costello et al. 2010).

Access rights better align users’ economic incentives with ecological goals (Essington et al. 2012). In an ITQ fishery, quota owners have incentives to maintain the targeted resource at a sustainable limit (ECR-1), and in some cases there is evidence of stock recovery (Hentrich and Salomon 2006). Chu (2009) reviewed 20 ITQ fisheries around the world finding that 12 stocks were improved after ITQ implementation, but they conclude that additional measures are still necessary to promote recovery, such as good enforcement and monitoring, or implementing ecosystem-based fisheries management (Chu 2009). ITQs can promote stewardship that enhances social resilience by instilling a longer-term perspective in resource management (SCR-3) and promoting decisions that maximize long-term profitability (Essington et al. 2012). For example, voluntary surveillance and monitoring institutions have been established under New Zealand ITQ fisheries (ECR-3) (Hentrich and Salomon 2006).

Multi-level governance can be possible, although ITQ systems are usually designed as top-down management institutions (SCR-4). An exception is the Netherlands ITQ system that is based on local management groups, and emerged from a co-management system between regional producer organizations (Hentrich and Salomon 2006). ITQs may not facilitate diversified livelihoods (SCR-2), for example fishers exiting New Zealand’s ITQ systems were found to lack any alternative livelihood option (citation). Fishing fleet mobility, given that rights are over a stock rather than over a territory, contributes to social resilience by allowing vessel operators to access fish stocks that vary in or shift their distribution so long as they do not shift outside of the jurisdiction of the ITQ system (SCR-5). Higher mobility is most likely in technology-intensive commercial fisheries, where larger operators may be able to change their operation in response to fish redistributions (Fulton 2011; Holbrook and Johnson 2014). However, there will be important constraints to this flexibility, related to competition for new areas and restrictions imposed by international borders (Cheung et al. 2012), as well as governance problems due to the changes in species’ distributions across national borders and the resulting implications for management institutions (Berkes 2006). Miller and Munro (2004) propose side payments between two countries as a way to increase ITQ flexibility under climate change when countries face an environmental regime shift and the access to the resource varies in time. Finally, and despite the fact that little progress has been made in incorporating community-based management into ITQ systems (Arnason 2005), the two approaches can be compatible (SCR-6), as observed for the case of the Tasmanian lobster fishery. There the success was due to a combination of ITQs with a strong tradition of participatory management (van Putten et al. 2013).

### Territorial Use Rights in Fisheries (TURFs)

Territorial Use Rights in Fisheries (TURFs) define property rights over spatial areas of the ocean, as opposed to rights over a portion of the catch (Costello et al. 2010). TURFs are fixed in space and provide concessions to individual fishers or unions/cooperatives to manage the resources. Monitoring and enforcement typically depends on the owners, while fisheries agencies are commonly responsible for establishing the overall total allowable catch (TAC). TURFs constitute a common near-shore fisheries management system in many countries such as Chile, Spain, Japan, and Mexico (Wilen et al. 2012).

TURF fisheries that are managed through incentive-based participation and effective enforcement may be better able to withstand climate impacts (Hilborn et al. 2003), and thus be more durable against environmental change. For example in Chile, TURFs resulted in increased abundance and size of managed and unmanaged species in comparison with open access areas (Gelcich et al. 2012; ECR-1). A survey of species and habitats inside and outside of several TURFs in Chile showed that reef fish species had significantly higher species richness, biomass, and density in TURFs compared with open access areas (ECR-2), even though these are not the managed species under property rights regimes (Gelcich et al. 2012). In Galicia, Spain the TURF system includes advisors working with fisher cooperatives, who facilitate and support decision-making processes and play a key role in communication between fishers, scientists, and policy makers (Macho et al. 2013). These strategies can in many cases allow for adaptive (SCR-1) and community-based management (SCR-6) (Hilborn et al. 2005; Defeo et al. 2014). Having spatial property rights may also create incentives for avoiding other anthropogenic pressures that lead to habitat destruction (ECR-3). In TURFs, fishers tend to more actively monitor their territories (Gelcich et al. 2008), protecting against sources of habitat destruction and pollution. Additionally, spatial property rights provide incentives for fishers to combat poaching, even giving fishers direct authority over surveillance. For example, in the Spanish Asturias TURF system for goose barnacle, members actively engage in surveillance to prevent illegal harvest (Rivera et al. 2014).

Since TURFs give exclusive control over the target species in a given area, they create incentives for broader ecosystem-wide stewardship (Costello et al. 2010), thus reducing fishing mortality and contributing to the ecological resilience of the fish populations (ECR-1) (Defeo et al. 2014). Additionally, social resilience is increased by maintaining the stock at desirable levels for a longer-term provision of services (SCR-3) (Defeo et al. 2014). Ownership of an area can also allow TURF owners to generate revenues through other compatible activities such as tourism, recreation, and aquaculture—thus diversifying local livelihoods and enhancing social resilience (SCR-2). For example, research has shown that TURFs in Chile have led to a high level of diversified income sources for the fishers involved (Moreno and Revenga 2012).

The governance of TURFS can include some top-down control but is generally community-based, which allows both for a participative bottom-up and multi-level governance structure involving the local cooperatives and/or unions of fishers and local and regional institutions in the governance process (SRC-4) (Ovando et al. 2013). The Asturias goose barnacle TURF system has promoted the incorporation of traditional knowledge into management and the matching of resource and management scales (Rivera et al. 2014). On-site decision-making also helps in designing management plans and rules that avoid perverse incentives such as the ‘race to fish’ or subsidizing overcapacity fleets and related industries (Pauly et al. 2002). For example in Japan, the institutional flexibility of the TURF system allowed for the bottom-up creation of new management associations for particular species when necessary (Wilen et al. 2012). Regarding fisher mobility, TURFs inhibit movement because access rights are to specific fishing grounds (e.g., Aburto et al. 2013). Their spatial nature causes TURFs to be potentially worse than ITQs, limited entry, or open access in terms of promoting fisher mobility, which could be quite problematic for resilience when fish stocks exhibit major shifts in distribution (SCR-5).

## Discussion

We propose a new framework for studying and addressing the question of resilience to climate change under different fishery regulatory regimes. We derive nine climate resilience criteria for fisheries, and use them to understand the potential socio-ecological benefits and deficiencies of four common fisheries systems. Our literature review does not provide a systematic comparison of how different systems perform in terms of resilience, although the criteria presented here could be applied in a quantitative comparison. Previous analyses have conducted systematic comparisons for specific dimensions of resilience (Leith et al. 2014; López-Angarita et al. 2014; Rivera et al. 2014), but our approach could facilitate more encompassing assessments of socio-ecological resilience. Additionally, we provide a framework for future research and suggest new questions based on the likely resilience effects, tradeoffs, and synergies revealed from our review of the existing literature (Table 2). The variation in resilience factors for different fisheries regulatory regimes is likely substantial. Design attributes of the regulatory regime (i.e., bottom-up vs. top-down management approaches; by-catch avoidance or mitigation measures; monitoring and enforcement) as well as the scale of the system may also play important roles in determining resilience to climate change. Nevertheless, we did find some general patterns in terms of resilience potential of the regulatory regimes examined here.

Open access appears to offer potential benefits in only one criterion, fisher mobility, although the consequence of fisher mobility can be counteractive, as explained before. There is little open access fishers can do in response to climate change beyond altering the quantity or location of effort following shifts in species distributions. Failure to build socio-economic resilience can result in important consequences, as climate change impacts act on the already overfished stocks that typify open access regimes. Furthermore, issues of migration, food security, and poverty that compromise resilience are also more likely to arise in open access settings (Allison and Ellis 2001).

Limited entry fisheries likely offer only modest gains in resilience compared to open access management—limited entry management can result in sustainable harvest, and the rules governing limited entry can be adapted to respond to changing conditions (i.e., adjusting seasonal closures to coincide with altered spawning times). But these benefits depend on the success of management institutions in achieving effective harvest controls, and restricting access alone often has limited benefits to stock status (Hilborn et al. 2003) while also failing to establish conditions for other socio-economic resilience factors.

ITQs have the advantage of providing stewardship incentives to quota owners, potentially resulting in more sustainable harvest (Costello et al. 2008; Grainger and Costello 2011). However, the social resilience of the fishery can be compromised as the implementation of the system of rights to owners may force excluded fishers to exit the fishery (Arnason 2005). Livelihood diversification options may be more limited to these former fishers who have lost access to the resource, as they have lost a source of income. On the other hand, the ability of quota holders to retain rights to access shifting stocks enhances resilience via their choice between pursuing target species in their new distribution or selling their permits to others willing to do so. However, shifts in species distribution may move beyond the current jurisdictional borders of the ITQ system, which together with high prices for transferability may pose important challenges in the future.

Spatial rights-based approaches such as TURFs appear to provide a better foundation for building resilience in fisheries by encouraging stewardship in fishers, as well as ecosystem-based management and conservation. As seen in Chilean and Japanese TURF systems, community and nested governance structures evolved due to the local nature of allocated fishing areas—producing a system that may be better able to respond to climate impacts. On the other hand, climate impacts and spatial rights could be highly spatially correlated, with negative climate impacts across a system of TURFs posing serious difficulties to the system’s resilience. In such a case, low mobility for fishers may decrease social resilience and alternative management scales and/or cooperation agreements may be fundamental. However, although spatial rights may seem vulnerable to climate-induced species shifts, this perspective ignores the potential socio-ecological resilience benefits that TURFs may offer to vulnerable species that are pushed into TURFs at the leading edge of their shifting range as opposed to into open access areas. Conversely, there are no studies of the potential incentives for overharvesting species at the trailing edge of a distribution shift, given that TURF owners may not be willing to steward a resource that they are losing due to climate change (Pinsky and Mantua 2014).

TURF owners and quota holders with a direct longer-term interest in the persistence of a fishery may be more willing to implement climate change mitigation and adaptation strategies, such as connected TURF networks or multi-species ITQs, to maintain their investment rather than simply exploiting the remaining population to maximize short-term gain.

Our analysis suggests a variety of future research directions on climate resiliency and fisheries. For one, there are likely other contributing factors to socio-ecological resilience in fisheries that have yet to be identified or well studied. For example, recent research shows the effect of strong leadership contributing to the success of fishery management (Gutierrez et al. 2011), a factor that may also contribute to climate change resilience. Additionally, high social vulnerability due to poverty and other factors, together with compromised human rights for fishers in some areas, may be related to resilience as well, where more secure and less vulnerable fishers have more incentives to sustainably manage their resource (Allison et al. 2012).

Second, the interactions between social and ecological factors that determine resilience are still poorly understood. We attempt to map these tradeoffs and feedbacks, but additional studies and analysis are needed. Quantifying these interactions can illuminate interesting tensions in the criteria—for example, alternative livelihoods can offer income during periods when environmental conditions have resulted in low yields; however, other economic opportunities may also undermine incentives to steward the resource and maintain fishing as an economic opportunity. Furthermore, it is possible that combining different management or regulatory systems may produce significant socio-ecological climate resilience synergies (e.g., Sanchirico and Wilen 2002; Costello and Kaffine 2010). One example could be coupling TURFs with marine reserves (Afflerbach et al. 2014; Lester et al. 2016).

Third, it is important to note that while we do not distinguish among our criteria in terms of value or importance, certain socio-ecological climate resilience factors are likely more critical than others and their relative importance could be context specific. We found that the amount of evidence in the literature supporting each criteria varies greatly; for example, there is a wealth of information regarding the effect of sustainable harvest levels on fish population stability, but much less investigation on the effect of fisher mobility in providing resilience to climate impacts (Pinsky and Mantua 2014).

Lastly, there is a need for more empirical research on resilience in fisheries SES. Establishing clear and replicable metrics and monitoring fisheries SES responses to climate impacts are key needs to quantify the relative benefits of our derived socio-ecological resilience criteria and the overall impact of managing fisheries for resilience. Future research can apply this framework to a given fishery in order to understand how the different resilience factors interact and vary in importance in a context-dependent manner. Similarly, comparing quantitative assessments for fisheries with different management regimes will allow for a better understanding of how management institutions facilitate or hinder resilience under climate change.

## Conclusion

The climate change impacts already affecting the world’s oceans provide a compelling need to understand whether current fisheries management systems are resilient and how they are likely to fare under future conditions. While complex, a socio-ecological resilience approach to fisheries management can offer prescriptive approaches to buffer and enhance recovery from climate change impacts. Rights-based fisheries regimes (e.g., TURFs, ITQs) are likely to outperform open access in terms of many potential resilience benefits, all systems exhibit variation in socio-ecological resilience, and design details of the regulatory and management instruments are fundamental.

Our framework offers a first approach to evaluating impact of regulatory systems on fisheries resilience to climate change. This framework highlights the multiple dimensions of resilience in a fishery and the complexity in designing a regulatory regime that contributes to resilience. Further research is needed to identify the design features that makes each of the regimes more resilient to climate change (i.e., multi-level governance vs. top-down control rules, community-based management vs. centralized management, geographic scale of management, specific management regulations) and the importance of socio-ecological contextual variables of the system (e.g., biological attributes of the target species, fishing technology). As steadily rising greenhouse gas emissions continue to produce climate change impacts, fisheries management will need to address these complex dynamics to build resilient systems for the future.

## Electronic supplementary material

Below is the link to the electronic supplementary material.

Supplementary material 1 (PDF 217 kb)
