# Supplementary material for: Fisheries regulatory regimes and resilience to climate change
Source: Ambio. 2016 Nov 16;46(4):399–412. doi: 10.1007/s13280-016-0850-1 (PMC5385667; doi:10.1007/s13280-016-0850-1)
Supplement: Supplementary file 1 — Supplementary material 1 (PDF 217 kb) [file 13280_2016_850_MOESM1_ESM.pdf]

Ambio

Electronic Supplementary Material
This supplementary material has not been peer reviewed

Title: **Fisheries regulatory regimes and resilience to Climate Change**

Supplementary Materials

S1. Methods

The approach conducted in this perspective is a qualitative analysis where we review existing literature on fisheries adaptation, resilience and management, and conceptualize the links between social and ecological resilience and four regulatory regimes. Our framework combines different research areas, including fisheries management, adaptation literature, the SES literature[1], and ecological resilience in fisheries. We start by reviewing scientific grey and published literature to identify empirical and theoretical evidence on ecological and social resilience for fisheries. We did not perform a systematic search with specific keywords because many studies relevant for our approach, for example do not acknowledge 'resilience' or 'SES', but are still relevant for the review. From our review, we derive nine general resilience criteria for fisheries systems by prioritizing: 1) criteria that are common to both the resilience and the fisheries regulations and management literature, i.e., a factor that is shown to increase resilience, and also has been linked with successful management; and 2) criteria that can be applied to different spatial scales and to different social contexts.

For each of the criteria identified we indicate the rationale behind the resilience benefits and show background references (below and Table 1 in main text). Because few studies perform quantitative analysis on resilience, we are not able to give weights to each criterion although we acknowledge they may differ greatly in terms of their contribution to the systems'

[1] Note that we understand fisheries as SES and we elaborate a framework that accounts for the social and ecological dimensions of the system, however, we do not develop our framework along the modules and tiers of the SES approach (Ostrom, 2009). We believe this could be addressed in future approaches following the general framework presented here.

resilience. Ecological criteria are derived from studies showing fisheries resistance and recovery to climate change, and general ecological resilience to climate change (Perry et al., 2010; Levin and Lubchenco, 2008; Bernhardt and Leslie, 2013). Social criteria are derived from the fisheries adaptation literature, including economic and social measures for climate change adaptation (Sumaila et al., 2011; Grafton, 2009), economic resilience literature (Leith et al., 2014) and adaptive capacity in SES (Rivera et al., 2014; 2015; Cinner and McClanahan, 2009; Maldonado and Moreno-Sanchez, 2014; Lopes et al., 2011).

A conceptual framework is developed which includes the potential interactions between social and ecological resilience dimensions, and as a way to illustrate the complex relationships and trade-offs that arise among the resilience criteria. We conceptualize resilience criteria in two axes (social and ecological resilience), and discuss the likely interactions among criteria (Figure 1).

Finally, using our derived resilience criteria, we evaluate and compare four classes of fisheries regulatory systems (TURFS, ITQs, Limited Entry and Open Access) in terms of their inherent potential to promote climate resilience. We evaluate the potential of the regimes to address each of the resilience criteria based on the evidence found in the literature, and based on the performance of each of the regulatory regimes in existing fisheries. We illustrate when possible how the different resilience criteria have been affected by the regulatory regime, or how the criteria performs under each regulatory option, with fisheries examples (Table 2 in the main text).

S2. Socio-Ecological Resilience criteria

While much of the adaptation literature focuses on the reduction of vulnerabilities to impacts, an alternative approach focuses on sources of resilience that create robustness to uncertainty and maintain system flexibility necessary to respond to change (Nelson et al., 2007). The more complex concept of resilience renders measurement problematic, but is also more powerful in recognizing the system dynamics associated with climate impacts. Therefore, we focus on resilience, a subtle yet important distinction often confused even in the scientific literature (Miller et al., 2010; Nelson et al., 2007). Specifically, we identify the factors expected to enhance ecological and social resilience to climate change for fisheries. We identify these factors based on a literature review of theoretical and empirical research examining social and ecological resilience to climate change, for ocean systems and more specifically for fisheries. We also reviewed literature on fisheries adaptation to climate change, especially adaptation for the social and economic systems. We synthesize these into nine ecological and socio-economic climate resilience (ECR and SCR, respectively) criteria based on the evidence found.

ECR1: Sustainable & Age-Diverse Target Populations – there is strong evidence that managing fish populations for stock sizes at or above those that produce maximum sustainable yields increases the population's resilience to inter-annual and longer-term environmental variability, especially when the variability is unpredictable (Perry et al., 2010; Hsieh et al., 2006; Sumaila et al., 2011; Brander, 2009; Hilborn et al., 2003). Furthermore, managing stock sizes with a buffer against climate shocks can be effective insurance. For example, small pelagic species such as anchoveta fluctuate strongly in response to varying environmental conditions (Chavez et al., 2003). Ignoring this climate-induced flux can lead to collapse – as happened in conjunction with overfishing Peruvian anchoveta in the early 1970s (Pauly et al., 2002).

In addition to increased abundance, shifted age class distributions composed of older fish can also be more resilient to climate change. Fish stocks susceptible to population crash are often characterized by predominantly younger age classes and altered life history characteristics (Perry et al., 2010). Retaining older age classes in fish populations can result in individuals more experienced in and able to survive environmental variability (Ottersen et al.,

2006); as well as a more diverse genetic pool creating higher population resistance to change (Reusch et al., 2005).

ECR2: Conserving Biodiversity & Habitats – Fisheries resilience is increased by conserving the habitat, ecological communities, and processes that fish stocks depend upon. There is evidence that higher diversity promotes marine community robustness (Levin and Lubchenco, 2008). Preventing biodiversity loss can help ecosystems resist climate stressors by providing multiple food sources for higher trophic levels, and provides functional redundancy that can maintain community structure and trophic interactions in the face of short-term lowered abundance or loss of a species (Peterson et al., 1998). Gear restrictions such as limiting bottom trawling can prevent habitat destruction, maintain ecosystem heterogeneity, and increase climate resilience (Bernhardt and Leslie, 2013; Sumaila et al., 2011). Direct interventions such as recovering or planting mangroves can have a direct impact on fisheries productivity by enhancing nursery habitat and also decreasing climate impacts from sea level rise or extreme storm events (Spalding et al., 2013).

ECR3: Managing Existing Stressors – while highly dependent on local context, combinations of multiple stressors can harm marine ecosystems more than the sum of individual stressors (Crain et al., 2008). This implies that reducing impact from overfishing, illegal fishing, pollution, habitat destruction, and other non-climate anthropogenic impacts may increase general system resilience, including resilience to climate change. For example, avoiding habitat destruction and impairment from coastal development, marine industrial use, and other stressors can indirectly buffer fisheries against climate change impacts by maintaining and enhancing carrying capacity and ecosystem function necessary for fish growth, reproduction, and survival (Halpern et al., 2008). Decreasing local sources of low pH input from wastewater and industrial outflows (Feely et al., 2010; Cai et al, 2011), as well as SOx and NOx deposition from sources such as coal-fired power plants, can mitigate local acidification of coastal waters (Kelly and Caldwell, 2013) and thus buffer against climate-induced acidification. However it is important to note that while these actions undoubtedly increase general system resilience, whether or not they are useful to

specifically increase *climate* resilience depends upon the current system state, magnitude of projected climate impacts, and interactions between stressors which are still poorly understood (Barange and Perry, 2009).

SCR1: Adaptive Management – Experimental, iterative management pre-designed to learn from successes and failures increases fishery resilience to climate change via enhanced capacity to respond to uncertain and unforeseen events. Increased environmental variability and uncertainty due to climate change means that abrupt and unexpected changes will occur to fish abundance, distribution and ultimately catch (Lehodey et al., 2006) – which could render management systems already struggling to cope with current conditions dysfunctional (Gibbs 2007; Botsford et al., 1997). Adaptive management can provide the flexibility to allow fisheries to respond to variable conditions – for example, the South African anchoveta and sardine purse seine fisheries are jointly managed, allowing the fishers to switch target species and harvest levels in response to the asynchronous effects of climate change on their respective abundances (de Moor et al., 2008). Adaptive, experimental management not only can effectively reduce uncertainty in stock management (Bostford et al., 1997), but it can also promote the institutional learning and flexibility necessary to cope with the uncertainty and surprise inherent in climate change (Tompkins and Adger 2005; Kofinas and Chapin, 2009). Given the difficulties in evaluating fish stocks, combined with the uncertainties in local climate change and the poorly known interactive effects of climate impacts with other stressors, the ability to incorporate learning and new science into an adaptive management framework is likely to enhance fishery resilience to climate change (Plaganyi et al., 2011; Kell et al., 2005).

SCR2: Diversified livelihoods – The more alternative employment activities within a fishing community, the more resilient the community will likely be to shocks associated with climate change (Grafton, 2009; Allison and Ellis, 2001; Sumaila et al., 2011). Fortunately from a resilience perspective, small-scale fishers frequently exhibit occupational multiplicity, or the use of several income-generating activities such as aquaculture, tourism, and involvement in monitoring and management. They also are characterized by occupational mobility,

geographical mobility (fisher migration), and diversification within the fisheries sector (e.g. targeting multiple species, using several gear types) (Badjeck et al., 2010). Those with the ability to switch between occupations may be more amenable to fishery closures that may be required to recover stocks following a climate shock (Cinner, 2007), although shifts in livelihood may result in unsustainable pressures on other resources (Badjeck et al., 2010; Brashares et al., 2004). However, highly dependent users, for which poverty makes resource use obligatory to survival, will be less adaptive than those with a lower discount rate for the resource (Leith et al., 2014; Cinner et al. 2011; Allison et al. 2009). As noted by Allison and Ellis (2001) in several case study fisheries, livelihood diversification and fisher mobility can work as substitute sources of resilience for the community.

SCR3: Promoting Long Term Stewardship – Stewardship refers to the protection and sustainable use of the environment and its resources. Fisheries stewardship is important in order to maintain sustainable fish stock levels for consistent and predictable catches. Assigning fishers property rights over a physical space or over a share of the stock are means of increasing stewardship incentives (Costello et al. 2008; Essington et al., 2012) – especially when the rights holders internalize the environmental and economic costs of using destructive or non-selective fishing gear by, for example, avoiding non-target species, or account for the longer term impacts resulting from choosing specific fishing locations (Cancino et al., 2007; Jardine and Sanchirico, 2012). Additionally, long-lasting and secure access can promote investment in research to improve the precision of stock assessments that are used to establish annual catch limits (Branch, 2009). Owners of access rights may also advocate for lower catch levels in the face of uncertain stock status (Branch, 2009; Essington et al., 2012), thereby reducing fishing mortality and contributing to ecological resilience of the fish populations and socio-economic resilience of the owners. Without ownership creating a direct interest in the future value of a fishery, fishers may sacrifice long-term productivity for short-term profits and pressure managers into setting catch limits that are too high (Nowlis and van Benthem, 2012). Promoting stewardship has been shown to produce other resilience benefits such as incorporating ecosystem-based management and avoiding non-climate stressors (Gelcich et al., 2008).

SCR4: Multi-level Governance – A nested or multi-level governance model, defined as the combination of different institutions jointly coordinating resource management over varying geographical or administrative scales, promotes collaborations by linking communities, government agencies and non-government organizations from the local to the regional and national scales. These governance systems allow for greater flexibility and the opportunity to experiment with alternative responses, and may also create redundancy that promotes resilience in socioeconomic systems (Grafton, 2009). Conflicts between recreational and commercial fishing were identified as a major threat for the systems governance of the Tasmanian rock lobster fishery, leading to a low resource-sharing resilience score in their analysis (van Putten et al., 2013). Multi-level governance can potentially help in conflict solving for resource sharing. Networks between institutions (government, industry, research and environmental NGOs) are important in the context of adaptive management of fisheries (Leith et al., 2014; Sandström and Rova 2010). These are necessary under the multi-scale impacts of climate change, where large institutions that manage fisheries at a very broad scale are likely to ignore local heterogeneity (e.g., spawning aggregations that are readily targeted to extinction); whereas local institutions focused on specific species or locations may not be able to plan for larger scale drivers of change (e.g., shifts in species' distributions) (Hughes et al., 2005). In fact, according to Grafton (2009), different layers of management and complementary rules in fisheries governance can also increase management options across local, regional and national scales. The collaboration of a diverse set of stakeholders operating at different social and ecological levels can promote an adaptive governance framework (Hughes et al., 2005), with direct implications for adaptive management (SCR1). Matching management, resource and resource users scales is fundamental for sustainable management (Rivera et al., 2014), as well as incorporating local traditional knowledge for decision making (Berkes et al., 2010).

SCR5: Fisher Mobility – Mobility refers to the capacity of the fishing fleet and fishers to access different fishing grounds, and includes their ability to do so based on management regulations. Highly mobile fishers, with an ability to catch different species using alternative gears over a wide geographical area, with better technology and with access to capital, will be more able to adapt to climate change (Grafton, 2009). Thus, more technically advanced fleets and fleets in

developed countries already operating in long distance fishing grounds may be at an advantage, as may be countries whose exclusive economic zones are not strictly continental and have offshore territories or strategic international arrangements (Holbrook and Johnson, 2014; Sumaila et al., 2011; Fulton, 2011). In contrast, small-scale fishing operators will be less resilient due to their long-term association with fishing practices that relate to their identity (Holbrook and Johnson, 2014) and will therefore be less likely to adopt new strategies that involve accessing new fishing grounds. Of course, increased mobility can also allow fishers to serially deplete stocks as they move to new fishing grounds, continuing to use unsustainable fishing practices on new stocks. This behavior is distinguished from the mobility for climate resilience, which allows fishers to adapt and respond to spatial and temporal variance in abundance and distribution of target species, but must be coupled with sustainable fishing practices to confer resilience.

SCR6: Community-based management – refers to the equity in management decisions when fishers are part of the decision-making process and governance is generally bottom-up. Access to and participation in the wider decision-making processes often enhances socio-ecological resilience (Gelcich et al., 2010; Adger, 2003; Tompkins and Eakin, 2012; Chapin et al., 2009). Successful community-based resource management (such as co-management arrangements) can enhance climate resilience of communities, as well as ecosystems, as it allows for self- and re-organization subsequent to impacts (Tompkins and Eakin, 2012). For example, fisher cooperatives often take direct conservation actions such as establishment of private marine protected areas (Ovando et al., 2013). In New Zealand, self-efficacy and sense of community were found to be good predictors of community resilience and increased capacity to respond to sudden change after a volcanic eruption (Paton et al., 2001). Community managed fisheries may be able to react faster to external changes and avoid excessive pressures (such as overharvesting) over impacted resources at the local level.

References SM

Adger, W.N. 2003. *Governing natural resources: Institutional adaptation and resilience*. Negotiating Environmental Change: New Perspectives from Social Science, 193-208.

Adger W.N., Dessai S., Goulden M., Hulme M., Lorenzoni I., Nelson, D.R., Naess, Lars O., Wolf J., Wreford A. 2009. Are there social limits to adaptation to climate change? *Climatic Change* 93: 335–354.

Allison, E.H., Ellis, F. 2001. The livelihoods approach and management of small-scale fisheries, *Marine Policy* 25(5): 377–388.

Allison, E.H., Perry, A.L., Badjeck, M.C., Adger, N.W., Brown, K., Conway, D., Halls, A.S., Pilling, G.M., Reynolds, J.D., Andrew, N.L., Dulvy, N. K. 2009. Vulnerability of national economies to the impacts of climate change on fisheries, *Fish and fisheries 10*(2): 173-196.

Badjeck, M.-C., Allison, E. H., Halls, A. S., Dulvy, N. K. 2010. Impacts of climate variability and change on fishery-based livelihoods, *Marine Policy* 34(3): 375–383.

Barange, M., & Perry, R. I. 2009. Physical and ecological impacts of climate change relevant to marine and inland capture fisheries and aquaculture, *Climate change implications for fisheries and aquaculture* 7.

Bernhardt, Joanna R., Leslie, Heather M. 2013. Resilience to Climate Change in Coastal Marine Ecosystems. *Annual Review of Marine Science* 5: 371–92.

Berkes, F., Colding, J., Folke, C. 2000. Rediscovery of traditional ecological knowledge as adaptive management. *Ecological Applications* 10(5):1251–62.

Bohnsack, J.A. 1998. Application of Marine Reserves to Reef Fisheries Management, *Australian Journal of Ecology* 23: 298-304.

Botsford, L.W., Castilla, J.C., Peterson, C.H. 1997. The Management of Fisheries and Marine Ecosystems, *Science* 277: 509-515.

Branch, T. A. 2009. How do individual transferable quotas affect marine ecosystems? *Fish and Fisheries* 10(1): 39–57.

Brander, K.M. 2009. *Fisheries and Climate*. In: John H. Steele, Karl K. Turekian, & Steve A. Thorpe (Eds.), Encyclopedia of Ocean Sciences (Second Edition) (pp. 483–490). Oxford: Academic Press.

Brashares, J, Arcese P,Sam, M, Coppolillo P, Sinclair A, Balmford A. 2004. Bushmeat hunting, wildlife declines, and fish supply in West Africa, *Science* 306: 1180–3.

Cai, W. J., Hu, X., Huang, W. J., Murrell, M. C., Lehrter, J. C., Lohrenz, S. E., Chou, W.C., Zhai, W., Hollibaugh, J.T., Wang, Y., Zhao, P., Guo, X., Gundersen, K., Dai, M., Gong G.C. 2011. Acidification of subsurface coastal waters enhanced by eutrophication, *Nature Geoscience 4*(11): 766-770.

Cancino J.P., Uchida H., Wilen J.E. 2007. TURFs and ITQs: collective vs. individual decision making, *Marine Resource Economics* 22(4):391.

Chapin, F. Stuart III, Kofinas, Gary P., Folke, Carl (Editors). 2009. Principles of Ecosystem Stewardship Resilience-Based Natural Resource Management in a Changing World. *Springer Science+Business Media*, LLC 2009.

Chavez, F. P., Ryan, J., Lluch-Cota, S. E., Ñiquen, M. 2003. From anchovies to sardines and back: multidecadal change in the Pacific Ocean, *Science* 299 (5604): 217-221.

Cheung, W.W., Lam, V.W., Sarmiento, J.L., Kearney, K., Watson, R.E.G., Zeller, D., Pauly, D. 2010. Large-scale redistribution of maximum fisheries catch potential in the global ocean under climate change. *Global Change Biology* 16(1): 24-35.

Cinner, J.E., 2007. Designing marine reserves to reflect local socioeconomic conditions: lessons from long-enduring customary management systems, *Coral Reefs* 26: 1035–1045.

Cinner, J.E., Folke, C., Daw, T., Hicks, C.C. 2011. Responding to change: Using scenarios to understand how socioeconomic factors may influence amplifying or dampening exploitation feedbacks among Tanzanian fishers, *Global Environ Change* 21 (1):7-12.

Costello, C., Gaines, S. D., Lynham, J. 2008. Can Catch Shares Prevent Fisheries Collapse? *Science* 321(5896): 1678–1681.

Crain, C. M., Kroeker, K., Halpern, B. S. 2008. Interactive and cumulative effects of multiple human stressors in marine systems, *Ecology Letters 11*(12): 1304-1315.

Doney, S. C., Ruckelshaus, M., Duffy, J. E., Barry, J. P., Chan, F., English, C. A., Galindo, H. M., Grebmeier, J.M., Hollowed, A.B., Knowlton, N., Polovina, J., Rabalais, N.N., Sydeman, W.J., Talley, L.D. 2012. Climate change impacts on marine ecosystems, *Marine Science*, *4*.

Drinkwater, K. F., Beaugrand, G., Kaeriyama, M., Kim, S., Ottersen, G., Perry, R. I., Pörtner, H.O., Polovina J.J., Takasuka, A. 2010. On the processes linking climate to ecosystem changes, *Journal of Marine Systems* 79(3–4): 374-388

Edwards, M., Richardson, A.J. 2004. Impact of climate change on marine pelagic phenology and trophic mismatch. *Nature* 430: 881-884.

Essington, T. E., Melnychuk, M. C., Branch, T. A., Heppell, S. S., Jensen, O. P., Link, J. S., Martell, S.J.D. A.M. Parma, Pope J.G., Smith, A. D. M. 2012. Catch shares, fisheries, and ecological stewardship: a comparative analysis of resource responses to a rights-based policy instrument. *Conservation Letters* 5(3): 186–195.

Feely, R. A., Alin, S. R., Newton, J., Sabine, C. L., Warner, M., Devol, A., Maloy, C. 2010. The combined effects of ocean acidification, mixing, and respiration on pH and carbonate saturation in an urbanized estuary. *Estuarine, Coastal and Shelf Science 88*(4): 442-449.

Fulton, E. A. 2011. Interesting times: winners, losers, and system shifts under climate change around Australia. *ICES Journal of Marine Science* 68(6), 1329–1342.

Gelcich, S., Godoy, N., Prado, L., Castilla, J. C. 2008. Add-on conservation benefits of marine territorial user rights fishery policies in central Chile. *Ecological Applications* 18(1): 273-281.

Gibbs, M. T. 2007. Network governance in fisheries, *Marine Policy* 32: 113-119.

Grafton, R.Q. 2009. Adaptation to climate change in marine capture fisheries. *Marine Policy* 34(3): 606–615.

Halpern, B.S., S. Walbridge, K.A. Selkoe, C.V. Kappel, F. Micheli, C. D'Agrosa, J.F. Bruno, K.S. Casey, C. Ebert, H.E. Fox, R. Fujita, D. Heinemann, H.S. Lenihan, E.M.P. Madin, M.T. Perry, E.R. Selig, M. Spalding, R. Steneck, R. Watson. 2008. A Global Map of Human Impact on Marine Ecosystems, *Science* 319 (5865): 948-952.

Hilborn, R., Quinn, T.P., Schindler, D.E., Rogers, D.E. 2003. Biocomplexity and fisheries sustainability. *PNAS* 100(11): 6564–6568.

Hoegh-Guldberg, O., Bruno, J.F. 2010. The Impact of Climate Change on the World's Marine Ecosystems. *Science* 328(5985): 1523–1528.

Holbrook, N. J., Johnson, J.E. 2014. Climate change impacts and adaptation of commercial marine fisheries in Australia: a review of the science. *Climatic Change* 124:703–715.

Hsieh, C.H., Reiss, C.S., Hunter, J.R., Beddington, J. R., May, R.M., Sugihara, G. 2006. Fishing elevates variability in the abundance of exploited species. *Nature 443*(7113): 859-862.

Hughes, T.P., D.R. Bellwood, C. Folke, R.S. Steneck, J. Wilson. 2005. New paradigms for supporting the resilience of marine ecosystems. *TRENDS in Ecology and Evolution* 20(7): 380-386.

Jardine, S. L., Sanchirico, J. N. (012. Catch share programs in developing countries: A survey of the literature. *Marine Policy* 36(6): 1242–1254.

Kell, L.T., Pilling, G.M., O'Brien, C.M. 2005. Implications of climate change for the management of North Sea cod (*Gadus morhua*). *ICES Journal of Marine Science: Journal du Conseil*, *62*(7): 1483-1491.

Kelly, R. P., Caldwell, M. R. 2013. Ten Ways States Can Combat Ocean Acidification (and Why They Should). *Harvard Environmental Law Review 37:57-104*.

Kofinas, G.P., Chapin III, F.S. 2009. Sustaining livelihoods and human well-being during social-ecological change. *Principles of Ecosystem Stewardship* (pp. 55-75). Springer New York.

Lehodey, P., Alheit, J., Barange, M., Baumgartner, T., Beaugrand, G., Drinkwater, K., Fromentin, J.M. Hare, S.R., Ottersen , G., Perry, R.I., Roy, C., van der Lingen, C.D., Werner, F. 2006. Climate variability, fish, and fisheries. *Journal of Climate 19*(20).

Leith, P., Ogier, E., Pecl, G., Hoshino, E., Davidson, J., Haward, M. 2014. Towards a diagnostic approach to climate adaptation for fisheries. *Climatic Change* 122: 44-66.

Levin, S.A. and Lubchenco, J. 2008. Resilience, Robustness, and Marine Ecosystem-based Management. *BioScience* 58(1): 27-32.

Miller, K., Charles, A., Barange, M., Brander, K., Gallucci, V.F., Gasalla, M.A., Khan, A., Munro, G., Murtugudde, R., Ommer, R.E., Perry, R.I. 2010. Climate change, uncertainty, and resilient fisheries: Institutional responses through integrative science, *Progress in Oceanography* 87(1–4), 338-346.

Mueter, F.J., Megrey, B.A. 2006. Using multi-species surplus production models to estimate ecosystem-level maximum sustainable yields, *Fisheries Research*, *81*(2), 189-201.

Nelson, D.R., W.N. Adger, K. Brown. 2007. Adaptation to Environmental Change: Contributions of a Resilience Framework, *Annual Review of Environment and Resources* 32:395–419.

Nowlis, J., Van Benthem, A. A. 2012. Do Property Rights Lead to Sustainable Catch Increases? *Marine Resource Economics* 27(1), 89–105.

O'Connor, M., J.F. Bruno, S.D. Gaines, B.S. Halpern, S.E. Lester, B.P. Kinlan and J.M. Weiss. 2007. Temperature control of larval dispersal and the implications for marine ecology, evolution, and conservation, *PNAS* 104(4): 1266-1271.

Ottersen, G., Hjermann, D., Stenseth, N.C. 2006. Changes in spawning stock structure strengthens the link between climate and recruitment in a heavily fished cod stock, *Fisheries Oceanography* 15 (3): 230–243.

Ovando, D.A., R.T. Deacon, S.E. Lester, C. Costello, T. Van Leuvan, K. McIlwain, C.K. Strauss, M. Arbuckle, R. Fujita, S. Gelcich, H. Uchida. 2013. Conservation incentives and collective choices in cooperative fisheries*. Marine Policy* 37:132-140.

Paton, D., Millar, M., Johnston, D. 2001. Community resilience to volcanic hazard consequences. *Natural Hazards* 24: 157–169.

Pauly, D., V. Christensen, S. Guénette, T.J. Pitcher, U.R. Sumaila, C.J. Walters, R. Watson, D. Zeller. 2002. Towards sustainability in world fisheries, *Nature* 418: 689-695.

Perry, A.L., Low, P. J., Ellis, J. R., Reynolds, J. D. 2005. Climate Change and Distribution Shifts in Marine Fishes, *Science* 308(5730): 1912–1915.

Perry, R.I., Schweigert, J. F. 2008. Primary productivity and the carrying capacity for herring in NE Pacific marine ecosystems. *Progress in Oceanography 77*(2): 241-251.

Perry, R.I., R.E. Ommer, M. Barange, F. Werner. 2010. The challenge of adapting marine social–ecological systems to the additional stress of climate change, *Current Opinion in Environmental Sustainability* 2(5–6): 356-363.

Peterson, G., Allen, C.R., Holling, C.S. 1998. Ecological resilience, biodiversity, and scale, *Ecosystems* 1(1): 6-18.

Plaganyi, E. E., Weeks, S. J., Skewes, T. D., Gibbs, M. T., Poloczanska, E. S., Norman-López, A., L.K. Blamey, M. Soares, Robinson, W. M. 2011. Assessing the adequacy of current fisheries management under changing climate: a southern synopsis, *ICES Journal of Marine Science: Journal du Conseil 68*(6): 1305-1317.

Poloczanska, E. S., Brown, C. J., Sydeman, W. J., Kiessling, W., Schoeman, D. S., Moore, P. J., Brander, K., Bruno, J.F., Buckley, L.B., Burrows, M.T., Duarte, C.M., Halpern, B.S., Holding, J., Kappel, C.V., O'Connor, M.I., Pandolfi, J.M., Parmesan, C., Schwing, F., Thompson, S.A., Richardson, A. J. 2013. Global imprint of climate change on marine life, *Nature Climate Change 3*(10), 919-925.

Polovina, J.J., Howell, E.A., Abecassis, M. 2008. Ocean's least productive waters are expanding, *Geophysical Research Letters 35*(3).

Reusch, T. B., Ehlers, A., Hämmerli, A., Worm, B. 2005. Ecosystem recovery after climatic extremes enhanced by genotypic diversity. *PNAS 102*(8): 2826-2831.

Richardson, A. J., Brown, C. J., Brander, K., Bruno, J. F., Buckley, L., Burrows, M. T., Poloczanska, E. S. 2012. Climate change and marine life, *Biology letters 8*(6), 907-909.

Sandström A., Rova, C. 2010. Adaptive co-management networks: a comparative analysis of two fishery conservation areas in Sweden. *Ecology & Society* 15(3):14.

Spalding, M.D., McIvor, A.L., Beck, M.W., Koch, E.W., Möller, I., Reed, D.J., Rubinoff, P., Spencer, T., Tolhurst, T.J., Wamsley, T.V., Wesenbeeck, B.K, Wolanski, E., Woodroffe, C.D. 2013. Coastal ecosystems: a critical element of risk reduction, *Conservation Letters* 00(2013): 1–9.

Sumaila, U. R., Cheung, W. W., Lam, V. W., Pauly, D., Herrick, S. 2011. Climate change impacts on the biophysics and economics of world fisheries, *Nature Climate Change* 1(9), 449-456.

Tompkins E.L., Adger W.N. 2005. Defining response capacity to enhance climate change policy, *Environmental Science & Policy* 8: 562-571.

Tompkins, E.L. Eakin, H. 2012. Managing private and public adaptation to climate change, *Global Environmental Change* 22(1): 3-11.

van Putten, I. E., Jennings, S., Frusher, S., Gardner, C., Haward, M., Hobday, A.J., Nursey-Bray, M., Pecl, G., Punt, A., Revill. H. 2013. Building blocks of economic resilience to climate change: a south east Australian fisheries example. *Regional Environmental Change* 13(6): 1313-1323.
